# Voice cues are used in a similar way by blind and sighted adults when assessing women’s body size

**DOI:** 10.1038/s41598-017-10470-3

**Published:** 2017-09-04

**Authors:** Katarzyna Pisanski, David Feinberg, Anna Oleszkiewicz, Agnieszka Sorokowska

**Affiliations:** 1 Institute of Psychology, University of Wroclaw, Wroclaw, Poland; 2 Mammal Vocal Communication and Cognition Research Group, University of Sussex, Brighton, UK; 3 Department of Psychology, Behaviour & Neuroscience, McMaster University, Hamilton, Canada; 4 Smell & Taste Clinic, Department of Otorhinolaryngology, TU Dresden, Germany

## Abstract

Humans’ ability to gauge another person’s body size from their voice alone may serve multiple functions ranging from threat assessment to speaker normalization. However, how this ability is acquired remains unknown. In two experiments we tested whether sighted, congenitally blind and late blind adults could accurately judge the relative heights of women from paired voice stimuli, and importantly, whether errors in size estimation varied with task difficulty across gr﻿oups. Both blind (*n* = 56) and sighted (*n* = 61) listeners correctly judged women’s relative heights on approximately 70% of low difficulty trials, corroborating previous findings for judging men’s he﻿ights. However, accuracy dropped to chance levels for intermediate difficulty trials and to 25% for high difficulty trials, regardless of the listener’s sightedness, duration of vision loss, sex, or age. Thus, blind adults estimated women’s height with the same degree of accuracy, but also the same pattern of errors, as did sighted controls. Our findings provide further evidence that visual experience is not necessary for accurate body size estimation. Rather, both blind and sighted listeners appear to follow a general rule, mapping low auditory frequencies to largeness across a range of contexts. This sound-size mapping emerges without visual experience, and is likely very important for humans.

## Introduction

Voice-based body size estimation facilitates speaker normalization, allowing listeners to recognize speech sounds produced by people with vocal tracts of widely varying lengths^1^. The capacity to judge an indvidual’s size from their voice also has clear evolutionary functions for humans^2^, as for other mammals^3^. Yet how this ability develops in humans remains largely unknown.

Previous studies have focused almost exclusively on vocal communication in men as opposed to women, as sexual selection is thought to have operated more strongly on vocal indicators of putative formidability (e.g., body size, dominance and strength) in men than women^4^. These studies have shown that listeners can accurately judge the relative heights of men from their voices alone, particularly when the difference in height between two men is salient (i.e., exceeds 10 cm^5, 6^). Although it is known that listeners can accomplish this by relying on the formant frequencies of a speaker’s voice^5–9^ (resonances of the supralaryngeal vocal tract that correlate negatively with men’s body size^10^), exactly how listeners come to form this sound-size correspondence remains a mystery. The role of the fundamental frequency of the voice (perceived as voice pitch) in either aiding^5, 9^ or impairing^6^ listener’s estimates of body size also continues to be a topic of debate. Indeed, although listeners associate low voice pitch with large body size, voice pitch can explain less than 2% of the variance in men’s body size and less than 0.5% of the variance in women’s body size^10^.

There are several hypotheses regarding how humans attain the ability to estimate body size from the voice. Evidence that infants associate appropriate vocal patterns with size by four months of age^11^, and that blind listeners can estimates men’s relative body sizes with the same degree of accuracy as sighted listeners^12^, suggests that visual experience may not be necessary for accurate voice-based estimation of body size to emerge. In addition, evidence that fundamental and formant frequencies explain only a small portion of variance in height and weight within sexes^10^ further suggests that repeated audiovisual pairings of people’s voices with their bodies is unlikely to facilitate accurate size estimation.

As vision does not appear to play a vital role, this points to the possibility that humans may possess an evolved capacity to judge body size from the voice that is present at birth. However, this hypothesis is necessarily weakened by the fact that humans often make gross errors when assessing body size, particularly by associating low fundamental frequency with large size at the same-sex level^6^. An alternative hypothesis is that voice-based body size estimation follows a general perceptual bias, wherein listeners associate low frequency sounds with largeness whether assessing the size of inanimate objects, animals, or indeed, human bodies^6, 13–15^. This general rule of thumb would lead to accurate estimation of body size when a taller individual has perceptibly lower voice frequencies than a shorter individual. However, the rule is also likely to lead to errors when the opposite is true^6, 16^.

The present study is one of very few testing whether sighted listeners can accurately gauge women’s body size solely from their voice^6, 7, 17^, and is the first to test this capacity in blind persons. Listeners were presented with a series of voice pairs and selected which of the two voices belonged to the taller woman in the pair. We compared early blind, late blind, and sighted listeners to test the influence of visual experience and duration of vision loss on accuracy. Critically, we manipulated task difficulty across voice pairs. This allowed us to test the possibility that blind persons outperform sighted persons only on high-difficulty trials, where low-difficulty trials may show less variability in accuracy or ceiling effects (maximal performance). Manipulating task difficulty also allowed us to test whether visual experience predicts not only correct, but also incorrect size judgments. If blind listeners use similar mechanisms as sighted listeners to assess body size (e.g., always mapping low frequencies to largeness), we would expect both groups of participants to respond correctly on low-difficulty trials (where the taller women in voice pairs had relatively lower voice frequencies), and incorrectly on high-difficulty trials (where the taller women had relatively higher voice frequencies).

Thus, on the assumption that voice-based body size estimation follows a general perceptual rule mapping low frequencies to large size that requires no visual experience to emerge, we predicted that accuracy in listeners’ size assessments would decrease with task difficulty, and that late and early blind participants would perform no differently than sighted controls at each level of task difficulty.

## Experiment 1

Experiment 1 was designed to test the ability of sighted listeners to accurately estimate the relative body sizes of women of varying heights and voice frequencies. On the basis of these results we chose stimulus voice pairs of low, high and intermediate difficulty for use in Experiment 2 with blind and sighted listeners. We also measured the fundamental frequency (*F*0) and formant spacing (Δ*F*) of the stimulus voices (see Method).

### Results

**Figure 1:**
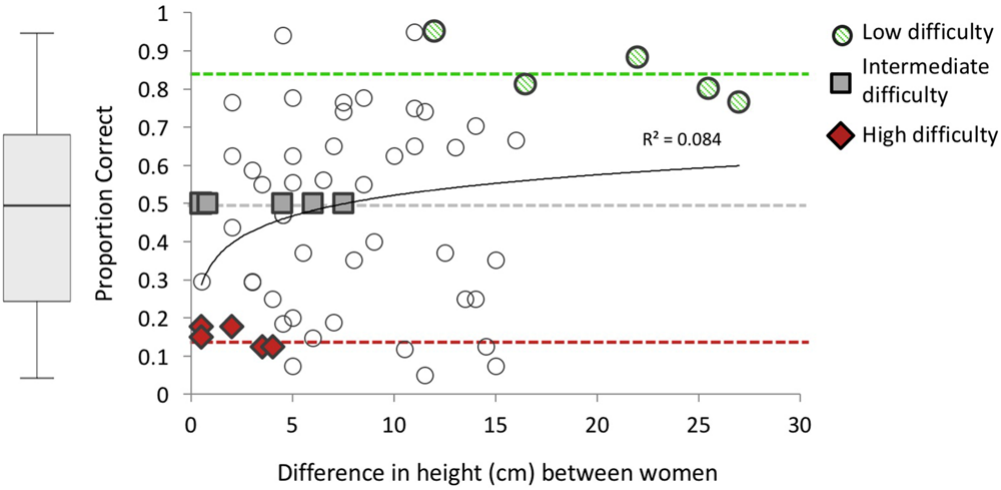
Experiment 1 proportion correct in sighted listeners’ estimates of women’s relative body size (mean ± SEM). Each point on the plot represents average accuracy scores for one given voice pair (based on *n* = 80 raters). From these, fifteen voice pairs of low, intermediate or high difficulty (labeled) were selected for use in Experiment 2. Dotted lines represent average accuracy scores for voice pairs at each level of difficulty (see also Table 1).

Eighty sighted men and women assessed the relative body sizes of women in 60 voice stimulus pairs. Responses in which the listener correctly chose the taller of two women were coded as ‘1’ and otherwise as ‘0’, and were then averaged within voice pairs to obtain a proportion correct accuracy score for each voice pair. No observations were excluded from analysis and all target variables have been reported. Accuracy scores were bi-modally distributed (Shapiro-Wilk *W* = 0.95, *p* = 0.02, *df* = 60; z-scored skewness = 0.08, kurtosis = −1.7) hence all statistical tests are nonparametric (two-tailed, $\propto$  = 0.05). Accuracy in listeners’ size estimates ranged widely from 5% to 95% across voice pairs (mean proportion. 48 ± 0.03 SEM, *n* = 60 pairs) and increased significantly with the difference in height between women in the given voice pair (Spearman’s *rho, r*_s_ = 0.29, *p* = 0.026). Accuracy scores plateaued and remained above chance when the difference in height between women reached approximately 15 cm (Fig. 1).

On the basis of these results, voice pairs of low (*n* = 5), intermediate (*n* = 5) and high (*n* = 5) difficulty were chosen for use in Experiment 2. Task difficulty was determined based on the accuracy of sighted listeners’ body size estimates, as well as the relative difference in height between the women in the voice stimulus pair. Thus, accuracy scores were above 75% for all low difficulty voice pairs (mean 84%), below 25% for all high difficulty pairs (mean 15%) and consistently at chance for all intermediate difficulty pairs (mean 50%) (Fig. 1). Table 1 presents mean differences in women’s heights and voice frequencies for voice pairs at each level of difficulty (see online supplementary material for data by voice pair). Note that for every low difficulty voice pair, the taller woman had substantially lower fundamental frequency (*F*0) and formant spacing (Δ*F*) than did the shorter woman, whereas the opposite was true for every high difficulty voice pair. For intermediate difficulty voice pairs the differences in women’s *F*0 and Δ*F* did not exceed perceptual discrimination thresholds^16, 18, 19^. Note also that the difference between women’s heights was considerably greater for low versus high difficulty voice pairs.

**Table 1:** Difference in height and voice frequencies between women in voice pairs at each level of difficulty (mean± *sd*; taller–shorter woman).

Level of Difficulty	*N* Voice Pairs	Difference in height (cm)	Difference in *F*0 (Hz)	Difference in Δ*F* (Hz)	Accuracy (Exp. 1)^a^
Low	5	20.4 ± 6.6	−29.32 ± 15.5	−51.79 ± 79.7	0.84 ± 0.07
Intermediate	5	4 ± 2.9	0.29 ± 10.2	8.64 ± 32.6	0.50 ± 0
High	5	2.2 ± 1.3	17.74 ± 18.8	43.34 ± 27.2	0.15 ± 0.03

^a^Proportion correct size estimates obtained from sighted listeners (*n* = 80) in Experiment 1.

## Experiment 2

Experiment 2 was designed to test whether blind adults can assess the relative body sizes of women with the same degree of accuracy as sighted adults, and whether error rates vary as a function of the difficulty of the task (determined on the basis of stimuli selected from Experiment 1), duration of vision loss, listener sex, and listener age.

### Results

The experiment included 61 sighted adult listeners with normal vision, 31 congenitally blind listeners, and 25 late-blind listeners (see Table 2). We used a Generalized Linear Mixed Model (GLMM) with binary logistic regression link to assess accuracy in listeners’ body size estimates. The dependent variable was the binary response (correct vs. incorrect estimation of relative body size). Sightedness (early blind, late blind, sighted) and level of task difficulty (low, intermediate, high) were included as fixed factors in the model, including both main and interaction effects and the intercept, and participant identity was included as a random subject variable. The initial GLMM included participant sex as a random variable and participant age as a covariate. Sex and age showed no significant effects and were therefore not included in the final model. All independent and dependent variables have been reported.

**Table 2:** Blind and sighted participant samples in Experiment 2.

Sample	Sex	*N*	Age		Sight Loss					
					Age of loss		Duration of loss (years)		Duration of loss (% of life)	
			*M*	Range	*M*	Range	*M*	Range	*M*	Range
Early blind	F	15	36.9	18–59	0.1	0–1.5	36.8	18–59	99.8	97–100
	M	16	32.0	17–50	0.2	0–1.5	31.8	17–50	99.2	93–100
Late blind	F	16	47.1	23–64	28.2	3–48	18.9	5–40	41.6	9.4–93
	M	9	51.3	29–61	33.9	4–53	17.4	1.5–50	32.2	4–93
Sighted	F	37	34.9	18–63						
	M	24	29.0	19–47						
All		117	36.5	17–64						

**Figure 2:**
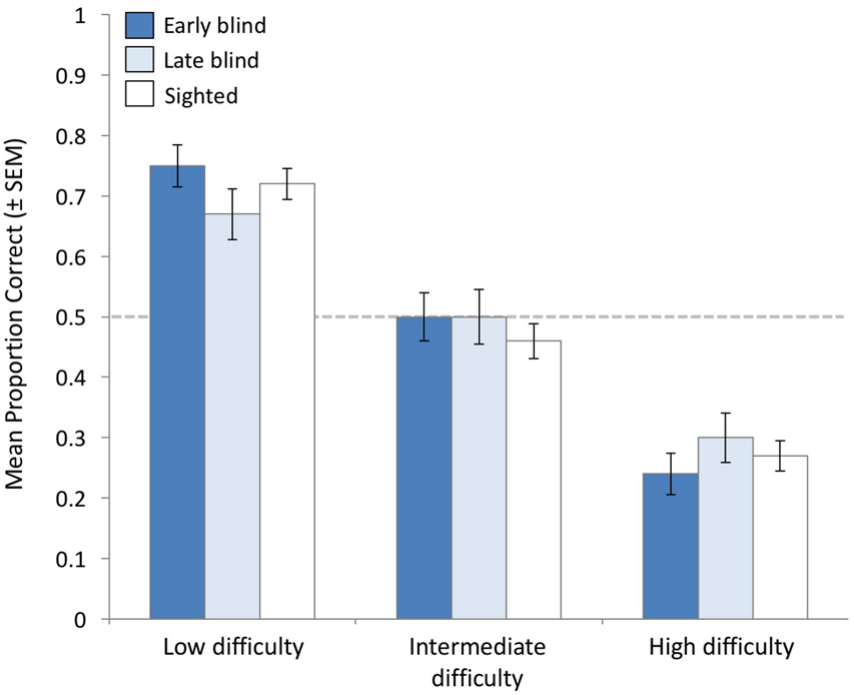
Experiment 2 proportion correct in sighted and blind listeners’ estimates of women’s relative body size (mean ± SEM). Accuracy varied as a function of task difficulty, with no differences among sighted, late blind, and early blind participants.

The GLMM was significant overall (intercept *F*_8, 1.7_ = 27.8, *p* < 0.001) and showed a significant main effect of task difficulty (*F*_2, 1.7_ = 93.7, *p* < 0.001). Critically, as clearly illustrated in Fig. 2, the model showed no main effect of sightedness (*F*_2, 1.7_ = 0.2, *p* = 0.89) and no interaction between sightedness and task difficulty (*F*_4, 1.7_ = 0.2, *p* = 0.35). The estimated marginal means in accurate body size estimation averaging across all participants were 73% correct for low difficulty voice pairs (95% CI 68.9–76.6, LSD test against chance controlling for multiple comparisons = 0.22, *p* < 0.001), 52% correct for intermediate difficulty voice pairs (CI 47.4–56%, LSD = 0.007, *p* = 0.68), and 28% correct for high difficulty voice pairs (CI 24.6–32.4%, LSD = −0.23, *p* < 0.001).

For blind participants, we then used binary logistic regressions to test whether duration of vision loss predicted accuracy in size estimates. Listeners’ binary responses (correct vs. incorrect size estimation) were included as the dependent variable, and duration of vision loss as a covariate. The models indicated that duration of vision loss in years (Wald ${\chi }_{1}^{2}$ = 0.57, *p* = 0.45) or as a percentage of life (Wald ${\chi }_{1}^{2}$ = 0.20, *p* = 0.65) had no effect on the accuracy of listeners’ size estimates. Examining early and late blind participants separately did not change this pattern of results.

## Discussion

Early and late blind adults estimated the relative heights of women from their voices with the same degree of accuracy, but also the same pattern of errors, as did sighted controls. For voice pairs of low difficulty, both blind and sighted listeners accurately indicated the taller of two women on approximately 70% of trials, whereas accuracy dropped to chance levels for voice pairs of intermediate difficulty and plummeted to 25% for pairs of high difficulty. Accuracy did not depend on how long blind persons had been blind (ranging from 1 to 59 years or 4% to 100% of their lives). Our results provide new insight into the perceptual mechanisms used by listeners to estimate body size from the voice, namely the strong influence of sound-size correspondences.

We demonstrate here that although blind persons can accurately estimate body size from the voice, this perhaps surprising capacity is in fact subject to the same pattern of errors as those observed in sighted listeners. Like sighted persons, blind persons accurately estimated women’s relative heights only when height differences exceeded 10 cm and the taller woman had relatively lower voice fundamental and formant frequencies. For high difficulty voice pairs, both blind and sighted participants consistently and incorrectly chose the shorter woman in the voice pair as taller. Thus, in addition to demonstrating that visual experience is not necessary for accurate body size estimation to emerge in adulthood, our findings suggest that body size estimation among both sighted and blind adults is based on a simple and general perceptual rule: low frequencies are mapped to large size.

A cross-modal correspondence between low frequencies and large size helps to explain how blind persons can judge body size from the voice with above-chance accuracy having never seen a human body, as taller men and women are likely to have relatively lower-frequency voices than shorter men and women^10^. At the same time, such a bias is known to lead to errors in body size estimation when exceptions to the rule arise, as previously demonstrated with sighted listeners^6, 20^, and with blind and sighted listeners in the present study. It is possible that blind persons learn to estimate body size from the voice through non-visual cross-modal correspondences, such as associating the frequency of a person’s voice with the spatial height from which it is projected. However, the weak relationship between voice frequencies and height within-sexes^10^, and recent evidence that low voice pitch can override elevation cues to height in voice-based judgments of body size (among sighted individuals)^20^, suggests that this mechanism is unlikely.

Although researchers have focused much more intensively on vocal communication of body size among men compared to women, previous studies suggest that sighted listeners use both fundamental and formant frequencies to assess women’s size^6, 17^ (as well as women’s femininity and attractiveness^21–25^) from the voice. These vocal characteristics are important in the social communication of both sexes. However, estimating women’s body size from the voice is likely to be more difficult than estimating men’s due to comparatively weaker relationships between women’s vocal parameters and body size^10^, and a wider harmonic spectrum in women’s voices that limits fine-grained resolution of formant-based cues to size^5, 9^. Indeed, playback studies using female voice pairs of variable relative heights show that sighted listeners perform just above chance when estimating women’s relative body size^6, 7, 17^. In our study, we show that estimates of women’s size can in fact be highly accurate when task difficulty is low.

Our findings challenge the hypothesis that blind persons posses ‘supra-normal’ auditory perception owing to compensatory plasticity of the brain^26^, at least in respect to voice-based social attributions. Previous studies suggest that blind persons, sometimes specifically early-blind^27^, perform better than sighted persons in low-level auditory tasks such as locating a sound in space^28^ or judging the direction of pitch change between two simple tones^29^. In this latter task, early blind participants outperformed late-blind and sighted participants across all levels of task difficulty. Duration of blindness predicted performance, suggesting a critical period for neural plasticity^29^. In contrast, our results show that duration of vision loss does not affect voice-based size estimates at any level of task difficulty. Other recent studies have likewise found no differences between blind and sighted participants in voice-based judgments of men’s body size^12^, trustworthiness, warmth and competence^30^. Taken together, this suggests that any advantage in auditory processing conferred to blind persons may not generalize to socially-relevant nonverbal voice tasks. There may be several reasons for this. Unlike tones, vocalizations are acoustically complex, broadband signals that are selectively processed in higher-level regions of the auditory cortex^31–33^, and by evoking social cognition, may activate a diverse range of neocortical regions.

In summary, our results provide evidence that a lifetime of visual experience does not contribute substantially, if at all, to voice-based judgments of women’s body size, and support the hypothesis that body size estimation is based on a general and deeply engrained perceptual rule, wherein low frequencies are associated with largeness in a wide range of domains and contexts^6, 13–15^.

## Method

### Experiment 1

#### Voice Stimuli

Thirty women (mean age 18.3 ± 1.1 years, height 164.3 ± 7.6 cm) were audio recorded speaking the monophthong vowels /α//i//ε//o//u/ (International Phonetic Alphabet) using a Sennheiser MKH 800 cardioid condenser microphone in an anechoic sound attenuated booth. Audio was digitally encoded with an M-Audio Fast Track Ultra interface at a sampling rate of 96 kHz and 32-bit amplitude quantization, and stored onto a computer as PCM WAV files using Adobe Soundbooth CS5 version 3.0. Vowel sounds were flanked by 250 ms of silence and each voice stimulus was amplitude normalized to 70 dB RMS SPL using Praat software^34^. Voice stimuli were then randomly paired to create 60 unique voice pairs in which the difference in height between women ranged from 1 to 27 cm (mean 8.2 ± 6 cm).

We measured women’s fundamental frequency (*F*0) and formant spacing (Δ*F*) using well-established methods^10, 35^. Briefly, mean *F*0 was measured using Praat’s autocorrelation algorithm with a search range of 100–600 Hz, and formants *F*1–*F*4 were measured using the Burg Linear Predictive Coding (LPC) algorithm, from which we computed mean formant spacing (Δ*F*)^35^. All measurements were performed separately on each vowel and then averaged across vowels within vocalizers.

#### Participants and Procedure

Eighty sighted men and women (mean age 18.7 ± 1.4 years) assessed the relative body sizes of women in the 60 voice pairs. On each trial, participants were presented with a pair of women’s voices, each speaking the full series of five vowels, and selected which of the two voices belonged to the taller woman. Voices were presented using a custom computer interface and through Sennheiser HD 280 professional headphones in a fully randomized order. The research was approved by the McMaster Research Ethics Board and was carried out in accordance with the provisions of the World Medical Association Declaration of Helsinki.

### Experiment 2

#### Participants

One hundred and twenty-three participants took part in the experiment, none of who participated in the first experiment. Six participants who reported hearing difficulties were excluded from the final dataset; no other observations were excluded from analysis. Our final sample included 56 blind men and women without any residual vision or light perception and 61 sighted men and women with normal vision. All participants were healthy with normal hearing and without neurological impairments. Groups were roughly matched on sex and age. Blind participants were classified as early blind if they were born congenitally blind (*n* = 28) or had lost their sight before age 2 (*n* = 3)^36^, otherwise they were classified as late blind (lost sight between age 3 and 53, *n* = 25). Duration of sight loss ranged from 1 to 59 years or 4% to 100% of the participant’s lifetime. Sample sizes were chosen on the basis of previous studies examining accuracy in voice-based body size estimation (e.g., refs 5 and 6) including a recent study comparing blind and sighted participants’ estimates of men’s body size^12^. All participants provided informed consent and were compensated for their participation. See Table 2 for additional sample characteristics, and online supplementary material for causes of vision loss.

#### Procedure

Participants completed the experiment in individual sessions following a procedure similar to that of Experiment 1. Thus, participants were presented with a single voice pair on each trial and selected which of the two voices in the pair belonged to the taller woman. Participants estimated the relative body sizes of women in all 15 voice pairs (low, intermediate and high difficulty), presented in a fully randomized order via a custom computer interface and through Sennheiser HD 201 professional headphones. To induce identical testing conditions for blind and sighted participants, all participants were fitted with a Mindfold mask (Mindfold Inc., Colorado, USA) that eliminated all incoming light and visual input without forcing the eyes closed, and their responses were provided orally and inputted by the experimenter. The research was approved by the University of Wroclaw Ethical Review Board and was carried out in accordance with the provisions of the World Medical Association Declaration of Helsinki.

## Electronic supplementary material

Supplementary Dataset 1
